# Non-Coding RNAs in Pancreatic Cancer Diagnostics and Therapy: Focus on lncRNAs, circRNAs, and piRNAs

**DOI:** 10.3390/cancers13164161

**Published:** 2021-08-19

**Authors:** Yiwei Li, Mohammed Najeeb Al Hallak, Philip A. Philip, Asfar S. Azmi, Ramzi M. Mohammad

**Affiliations:** Departments of Oncology, Karmanos Cancer Institute, Wayne State University School of Medicine, Detroit, MI 48201, USA; liyi@karmanos.org (Y.L.); alhallakm@karmanos.org (M.N.A.H.); philipp@karmanos.org (P.A.P.); azmia@karmanos.org (A.S.A.)

**Keywords:** ncRNA, lncRNA, circRNA, piRNA, pancreatic cancer

## Abstract

**Simple Summary:**

Pancreatic cancer is the seventh leading cause of cancer related death worldwide. In the United States, pancreatic cancer remains the fourth leading cause of cancer related death. The lack of early diagnosis and effective therapy contributes to the high mortality of pancreatic cancer. Therefore, there is an urgent need to find novel and effective biomarkers for the diagnosis and treatment of pancreatic cancer. Long noncoding RNA, circular RNAs and piwi-interacting RNA are non-coding RNAs and could become new biomarkers for the diagnosis, prognosis, and treatment of pancreatic cancer. We summarize the new findings on the roles of these non-coding RNAs in pancreatic cancer diagnosis, prognosis and targeted therapy.

**Abstract:**

Pancreatic cancer is an aggressive malignance with high mortality. The lack of early diagnosis and effective therapy contributes to the high mortality of this deadly disease. For a long time being, the alterations in coding RNAs have been considered as major targets for diagnosis and treatment of pancreatic cancer. However, with the advances in high-throughput next generation of sequencing more alterations in non-coding RNAs (ncRNAs) have been discovered in different cancers. Further mechanistic studies have demonstrated that ncRNAs such as long noncoding RNAs (lncRNA), circular RNAs (circRNA) and piwi-interacting RNA (piRNA) play vital roles in the regulation of tumorigenesis, tumor progression and prognosis. In recent years, increasing studies have focused on the roles of ncRNAs in the development and progression of pancreatic cancer. Novel findings have demonstrated that lncRNA, circRNA, and piRNA are critically involved in the regulation of gene expression and cellular signal transduction in pancreatic cancer. In this review, we summarize the current knowledge of roles of lncRNA, circRNA, and piRNA in the diagnosis and prognosis of pancreatic cancer, and molecular mechanisms underlying the regulation of these ncRNAs and related signaling in pancreatic cancer therapy. The information provided here will help to find new strategies for better treatment of pancreatic cancer.

## 1. Introduction

**Figure 1:**
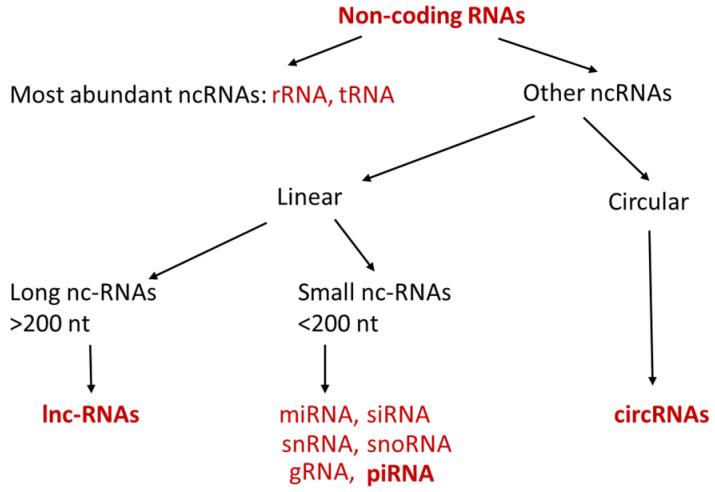
Types of ncRNAs. ncRNAs can be categorized into linear (long and small) and circular ncRNAs. ncRNAs in bold are discussed in the below sections.

Non-coding RNAs (ncRNAs) are short or long single strands of linear or circular RNA molecules, which have no ability to be translated into proteins [1,2,3,4]. The most abundant ncRNAs are ribosome RNA (rRNA) and transfer RNA (tRNA), which are critically involved in the process of translation and serve as house-keeping gene. Other ncRNAs can be categorized into long non-coding RNA (lncRNA) and small non-coding RNA according to their length (Figure 1). Small non-coding RNAs are usually shorter than 200 nt (nucleotides) and include microRNA (miRNA), small interfering RNA (siRNA), Piwi-interacting RNA (piRNA), small nucleolar RNA (snoRNA), small nuclear RNA (snRNA) and guide RNA (gRNA). Mechanistic studies have shown that these small ncRNAs play important roles in the regulation of transcription, post-transcription, and translation [2,3,4]. lncRNAs are RNA molecules which have lengths exceeding 200 nt and are not translated into proteins. One of the important functions of lncRNAs is epigenetic, transcriptional, and the post-transcriptional regulation of gene expression [5]. In addition to these long and small linear ncRNAs, there is another type of ncRNA molecule which forms a covalently closed loop, and it is named circular RNA (circRNA). The size of circRNA can range from under 100 nucleotide to over 4 kilobases. Similar as miRNAs and lncRNAs, circRNAs have recently received much attention in the field of cancer research because of their abilities to regulate gene expression [6,7,8].

## 2. Biogenesis and Functions of lncRNA, circRNA and piRNA

### 2.1. lncRNA

**Figure 2:**
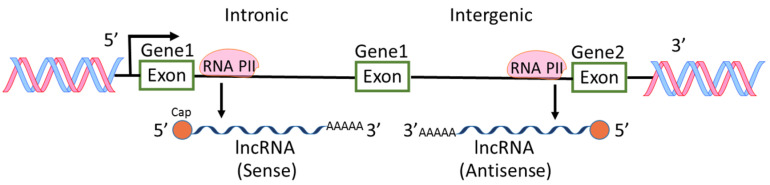
Biogenesis of lncRNAs. lncRNAs are transcribed by RNA polymerase II (RNA PII) intronically and intergenically. The sequences of lncRNAs are either sense or antisense compared to protein-coding genes.

Among the ncRNAs discovered, miRNAs are the most intensively studied and the roles of miRNAs in physiological and pathological regulations are extensively reported [6,7,9]. However, lncRNAs are also widely investigated because aberrant expression levels of lncRNAs are frequently found in various types of cancers [10,11,12]. H19 RNA was the first reported lncRNA in mammalian genome [13], followed by other lncRNA such as X-inactive-specific transcript [14]. In an earlier international collaborative study on mouse cDNAs, it was found that 11,665 cDNA are new non-coding messages and these ncRNAs represent a major component of the transcriptome [15]. From then on, lncRNAs were recognized and more lncRNAs were found through high-throughput genome analysis. Most lncRNAs are transcribed by RNA polymerase II, polyadenylated at 3′ end, spliced or un-spliced and capped at 5′ end [12,16] (Figure 2). The sequences of lncRNAs are either sense or antisense compared to protein-coding genes. The locations of lncRNA sequences are within introns or intergenic regions of genes. lncRNAs have diverse cellular functions including transcriptional regulation, epigenetic gene regulation, chromatin remodeling, cis and trans gene expression, etc. [5,12,17,18]. Because of the diverse functions of lncRNAs, the aberrant levels of lncRNAs in pathological status including cancers and inflammations have received much attention in the field of cancer research [2,5,19].

### 2.2. circRNA

**Figure 3:**
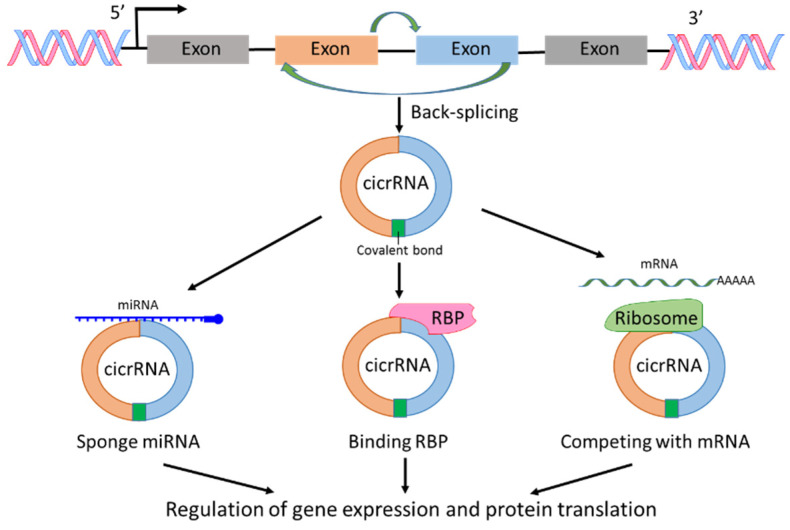
Biogenesis and functions of circRNAs. circRNA is produced by back-splicing and the fragments of RNA form a closed circular nucleotide structure connected by covalent bond. circRNAs interact with RNA binding protein (RBP), sponge with miRNAs and compete with mRNAs, leading to regulation of gene expression and protein translation.

circRNAs are a sub-group of ncRNAs and play significant roles in the regulation of transcription and post-transcription [20,21]. The first report on circRNA was published 40 years ago [22]. In the report, viroids were recognized as covalently closed circular RNA molecules. circRNAs are derived from pre-mRNAs; however, circRNAs lack the ability as mRNA to translate to protein. circRNAs can be generated from exons, introns, intergenic regions, 3′-UTR or 5′-UTR, but most circRNAs are derived from exons through spliceosomal and other machineries [20,21,22,23,24] (Figure 3). The investigations on circRNA production showed that RNA splicing such as exon skipping and back-splicing is important for circRNA creation [20,21,22,23,24]. The fragments of RNA created by RNA splicing form a closed circular nucleotide structure connected by covalent bond (Figure 3). Because of the circular structure, circRNAs are more stable than linear mRNAs. With the development of RNA-sequencing technology, new circRNAs have been discovered rapidly. The mechanisms of circRNAs regulating cellular signaling could include interaction with RNA binding proteins (RBPs), sponge with miRNAs, and competing with endogenous RNAs or RNA splicing, leading to posttranscriptional regulation of gene expression and protein translation [20,21,23]. In addition, most circRNAs are evolutionarily conserved and stable [25]. Any alternations in the expression level of specific types of circRNAs could reflect a pathological state. Therefore, circRNAs could be used as biomarkers for cancer diagnosis and treatment, and are considered a hot topic in cancer research [7,8,26,27].

### 2.3. piRNAs

**Figure 4:**
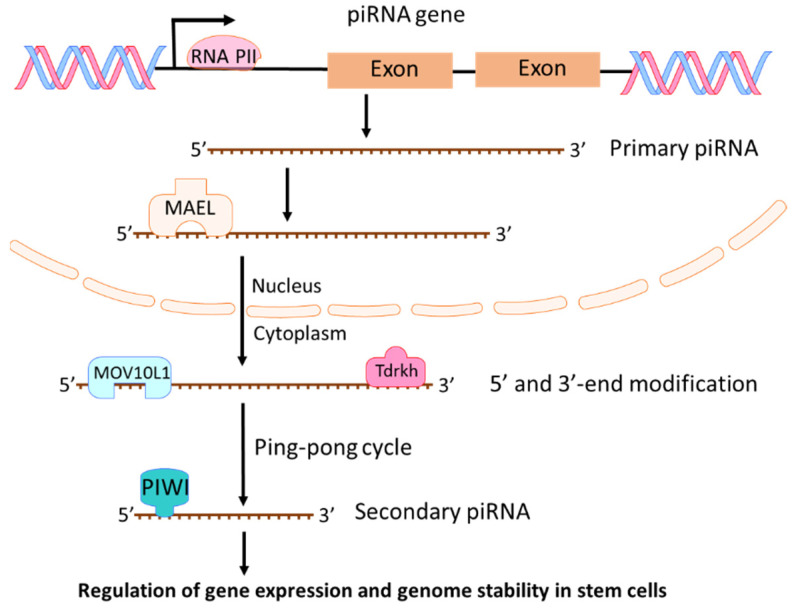
Biogenesis and functions of piRNAs. Primary piRNA is transcripted by RNA polymerase II (Pol II) from piRNA genes. By binding to MAEL, the primary piRNA is transported into cytoplasm where the primary piRNA undergoes 5′ and 3′-end modification and a ping-pong cycle to create secondary piRNAs. piRNAs can regulate gene expression and genome stability in stem cells.

piRNAs are a class of short (about 21 to 35 nucleotides) single-stranded RNAs. They interact with PIWI proteins to silence genetic elements such as transposons in the maintenance of genome stability and integrity [28,29,30,31]. Four PIWI proteins exist in humans: PIWIL1 (also known as HIWI), PIWIL2 (also known as HILI), PIWIL3 (also known as HIWI3), and PIWIL4 (as known as HIWI2) [32]. PIWI proteins play critical roles in the control of cell proliferation, apoptosis, cell movement, and genomic stability [32]. In 2006, piRNAs were first identified and named as “PIWI-interacting RNAs” in a mouse study on PIWI proteins and genome stability [33]. From then on, more piRNAs were identified based on their association with PIWI proteins and their biogenesis [34,35]. Two steps exist in the process of piRNA biogenesis [28]. First, a single-strand primary piRNA is transcripted by RNA polymerase II (Pol II) from a specific genomic location known as piRNA clusters (Figure 4). Through binding to a RNA-binding protein MAEL, the primary piRNA is transported into cytoplasm where the primary piRNA undergoes 5′ and 3′-end modification with MOV10L1 and Tdrkh [28,36,37], leading to increased stability and affinity to PIWI protein [38]. Then, primary piRNAs go through a ping-pong cycle to guide PIWI protein mediated cleavage of transcripts and create secondary piRNAs (Figure 4). The most investigated function of piRNA is its suppression of transposable elements (TEs) through association with PIWI proteins [28,39]. However, piRNAs could also regulate endogenous gene expression in stem cells during development [40,41]. piRNA regulates mRNA mainly through RNA degradation. piRNAs combined with MIWI (one of mouse PIWI proteins) could target mRNAs with imperfect base pairing and induce mRNA degradation through MIWI-dependent cleavage [42,43]. Although piRNAs play crucial roles in germ cells and adult stem cells, growing evidences have shown that piRNA and PIWI proteins are involved in tumorigenesis [44,45,46,47]. Recently, more investigations are focused on the roles of piRNAs in cancer development and progression [45,48,49,50,51]. Because piRNAs regulate gene expression and genome stability in stem cells, any deregulation of piRNAs could cause the development of cancer stem cells and promote cancer progression.

## 3. Major lncRNAs in Pancreatic Cancer

Because of the diverse biofunctions of lncRNAs, the roles of lncRNAs in pancreatic cancers can be oncogenic or tumor suppressive. Among the growing number of lncRNAs found in pancreatic cancers, in this section, we summarized some important lncRNAs which play critical roles in pancreatic cancer (Table 1).

### 3.1. HOTAIR

lncRNA HOTAIR (HOX transcript antisense RNA) is an oncogene in pancreatic cancer [52]. It was found that a higher level of HOTAIR had strong associations with susceptibility of pancreatic cancer [52]. A high expression of HOTAIR was also associated with proliferation and the metastasis of pancreatic cancers [55]. HOTAIR could transcriptionally regulate the expression of hundreds of genes in both PRC2-dependent and PRC2-independent manner in pancreatic cancer cells [53]. HOTAIR could also couple with EZH2 to silence tumor suppressor miR-34a in pancreatic cancer cells, thereby inducing cancer cell proliferation [54], suggesting the regulatory role of HOTAIR in EZH2/EMT pathway. Moreover, studies showed that HOTAIR enhanced pancreatic cancer resistance to gemcitabine and TNF-related apoptosis-inducing ligand, causing chemoresistance and progression of pancreatic cancer [56,57]. In addition, HOTAIR could also regulate the sensitivity of radiotherapy. Knockdown of HOTAIR enhanced the radiosensitivity of pancreatic cancer [95]. Furthermore, HOTAIR expression was associated with more aggressive pancreatic cancer, suggesting its prognostic role in pancreatic cancer [53].

### 3.2. MALAT1

Metastasis-associated lung adenocarcinoma transcript 1 (MALAT-1) is highly conserved and ubiquitously expressed lncRNA [96,97]. MALAT-1 was firstly discovered in non-small cell lung cancer [96,97]. However, high expression of MALAT-1 has been found in various cancer tissues including pancreatic cancer [63,98,99]; therefore, MALAT-1 is considered as an oncogenic lncRNA. In pancreatic cancer, increased expression of MALAT-1 promoted cell growth, migration, and invasion [59]. MALAT-1 also transcriptionally regulated Sox-2 expression and enhanced stem cell-like phenotypes in pancreatic cancer cells, suggesting its role in pancreatic stemness and tumorigenesis [60]. A study also showed that MALAT-1 promoted aggressive pancreatic cancer proliferation and metastasis via stimulation of autophagy [61]. MALAT-1 could also exert its oncogenic effects through mediation of EZH2, miR-216a, miR-217, miR-200c, or Hippo-YAP signaling in pancreatic cancers [58,62,64,65,66]. Therefore, MALAT1 regulates miRNAs, mRNAs, and proteins in stemness and autophagy pathways in pancreatic cancer. Importantly, overexpression of MALAT-1 has been correlated with advanced tumor stages, metastasis, and poor survival in pancreatic cancers [63,100].

### 3.3. GAS5

lncRNA growth arrest-specific 5 (GAS5) is a tumor suppressor found in various cancers including pancreatic cancer [101,102]. In pancreatic cancer, downregulation of GAS5 increased cell proliferation by regulating CDK6 transcriptionally, suggesting the tumor suppressive role of GAS5 [89]. GAS5 also reversed EMT, inhibited metastasis and increased the sensitivity of pancreatic cancer stem cells to gemcitabine through targeting miR-221/SOCS3 signaling [88]. Thus, GAS5 has a role in the regulation of cell cycle and EMT pathways. Studies also showed that GAS5 suppressed pancreatic cancer metastasis and chemoresistance through epigenetic regulation of miR-32 or miR-181c [86,87]. All findings demonstrate that GAS5 is a tumor suppressive lncRNA.

### 3.4. MEG3

MEG3 is another tumor suppressive lncRNA in cancers including pancreatic cancer [103]. The expression levels of MEG3 in both pancreatic cancer tissues and cells were found to be much lower than that in normal tissues and cells [92]. Knockdown of MEG3 promoted pancreatic cancer cell proliferation, migration, and invasion [92] while overexpression of MEG3 suppressed pancreatic neuroendocrine tumor cell viability, invasion, and migration [94]. The mechanisms underlying the tumor suppressive effects of MEG3 could be mediated through epigenetic regulation of c-Met. MEG3 was found to bind to unique genomic regions in and around c-Met gene and inhibit c-Met expression, leading to tumor suppression [91,93]. In addition, MEG3 could also exert its anti-cancer effects on pancreatic cancer by regulation of the PI3K/AKT signaling pathway [90]. Therefore, MEG3 affects multiple cellular signaling in pancreatic cancer.

### 3.5. H19

H19 is an oncogenic lncRNA which antagonizes tumor suppressive let-7 [104]. In pancreatic cancer, the expression of H19 was significantly increased and the overexpression of H19 was correlated with histological grade and invasion of pancreatic cancer [68]. Knockdown of H19 in pancreatic cancer cells inhibited cell proliferation and tumor growth with G0/G1 arrest and downregulation of E2F-1 transcription factor [68]. Knockdown of H19 also inhibited metastasis of pancreatic cancer [72]. It was found that H19 promoted pancreatic cancer cell invasion and migration by upregulation of HMGA2-mediated EMT through antagonizing let-7 [67], demonstrating the critical role of H19/let-7/HMGA2/EMT signaling axis in pancreatic cancer progression. In addition to the association of H19 with let-7, H19 could also correlate with miR-675 or miR194 to modulate EMT, cell proliferation, migration, and metastasis of pancreatic cancer [69,70,71], suggesting its role in the regulation of the EMT pathway.

### 3.6. PVT1

PVT1 is an oncogenic lncRNA [105]. In pancreatic cancer, higher expression of PVT1 was found in cancer tissues, and the high expression of PVT1 was positively correlated with poor survival of patients [73,76]. PVT1 promoted cell proliferation and migration through transcriptional and epigenetic regulation of p21 and miR-448 [78,80]. PVT1 also promoted autophagy and cell growth by regulating miR-20a-5p and ULK1 signaling, leading to the development of pancreatic cancer [74]. More importantly, PVT1 was identified as a regulator of gemcitabine sensitivity [77,79,81]. Functional inactivation of PVT1 resulted in the enhanced sensitivity to gemcitabine in pancreatic cancers [79]. In addition, PVT1 also promoted pancreatic cancer development through regulation of miR519, HIF-1, YKT6, RAB7, and VAMP3 [75,76], suggesting its diverse oncogenic effects with multiple signaling regulation.

### 3.7. HOTTIP

HOXA transcript at the distal tip (HOTTIP) is another oncogenic lncRNA [106]. HOTTIP is located at the 5′ end of HOXA cluster [106]. The expression of HOTTIP is increased in pancreatic cancer [84]. It was found that ectopic HOTTIP expression promoted growth and invasiveness in pancreatic ductal adenocarcinoma [84] and that HOTTIP modulated pancreatic cancer stem cell properties by regulating HOXA9 epigenetically [82]. More importantly, HOTTIP could also regulate drug resistance through regulation of miR-137 and HOXA13 [83,85], suggesting its role in HOXA pathway.

## 4. circRNAs in Pancreatic Cancer

In recent years, studies on circRNAs in cancer have become a hot topic in cancer research, especially after advanced next-generation sequencing technology was developed [7,21,107]. However, compared to other types of cancers, research on circRNAs in pancreatic cancer is still in its early stages. By conducting expression profiling of circRNAs using pancreatic cancer tissues and further mechanistic studies, it was found that some circRNAs are oncogenic while others are tumor suppressive in pancreatic cancers [108,109] (Table 2).

### 4.1. circRNA Expression Profiling in Pancreatic Cancer

In 2015, a group of investigators reported for the first time the microarray analysis of circRNA profiles in six pairs of pancreatic ductal adenocarcinoma and adjacent normal tissues [108]. They further analyzed the circRNA expression profile and identified a class of circRNAs that was responsible for tumorigenesis of pancreatic cancer. The top five up-regulated circRNAs in pancreatic cancers were hsa_circ_0072088, hsa_circ_0030235, hsa_circ_0001946, hsa_circ_0060055 and hsa_circ_0005397 whereas the top five down-regulated circRNAs were hsa_circ_0013587, hsa_circ_0075410, hsa_circ_0008768, hsa_circ_0080712, and hsa_circ_0000257 [122]. GO enrichment and pathway analysis showed that the most significantly altered circRNAs in pancreatic cancer were related to small GTPase regulator activity, Ras GTPase binding, RNA binding, and VEGF signaling [122], which have been known to be significantly altered in pancreatic cancer [123,124,125]. These findings suggest that these circRNAs could be molecular targets for the treatment of pancreatic cancer.

In 2016, another group of investigators conducted a microarray analysis of circRNA profiles in 20 pairs of pancreatic ductal adenocarcinoma and adjacent normal tissues and deposited the data in GEO database (GEO79634) [109]. The top 10 differently expressed circRNAs were identified in this study. Among them, five circRNAs (hsa_circ_102051, hsa_circ_102619, hsa_circ_104270, hsa_circ_102049 and hsa_circ_104227) were up-regulated while five circRNAs (hsa_circ_000167, hsa_circ_103809, hsa_circ_104700, hsa_circ_001846 and hsa_circ_102728) were down-regulated. They also found that a specific set of circRNAs (hsa_circRNA_100435, hsa_circRNA_103076, hsa_circRNA_103309, hsa_circRNA_000780, hsa_circRNA_101252, hsa_circRNA_102374, hsa_circRNA_104433, hsa_circRNA_104882, hsa_circRNA_101717, hsa_circRNA_104084, hsa_circRNA_100646 and hsa_circRNA_102213) could bind to miR-15a or miR-505 to modulate gene expression in pancreatic cancer [109]. miR-15a and miR-506 have been found to inhibit cell proliferation, EMT, and chemoresistance in pancreatic cancer [126,127].

Because circRNAs can function as miRNA sponges, the above mentioned circRNA profiling data from two sets of pancreatic tissues were further analyzed with miRNA profiling data. In the study, circRNA and miRNA interactions were predicted between differentially expressed circRNAs and miRNAs using computerized analysis [128]. A total of 51 interactions between circRNAs and miRNAs were found. Further analysis showed that mitogen-activated protein kinase, PI3K/AKT, and WNT/β-catenin signaling pathways were associated with the development of pancreatic cancer [128].

**Figure 5:**
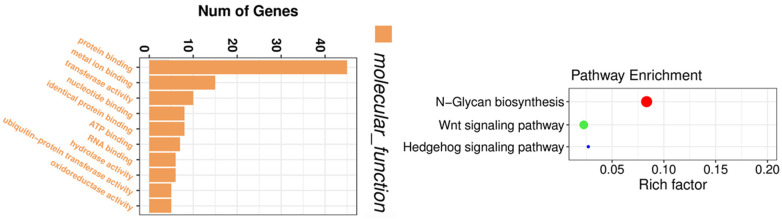
Gene enrichment analysis on circRNAs altered by thymoquinone. The altered circRNAs were associated with the binding of proteins, metal ions, nucleotide, ATP, and RNAs, and correlated with Wnt and Hedgehog signaling pathways.

In addition to the microarray technique used for circRNA profiling, high-throughput circRNA-sequencing has also been utilized for circRNA expression profiling [129,130]. We have conducted a circRNA-sequencing experiment to obtain the circRNA expression profiles before and after thymoquinone treatment in pancreatic cancer cells. By circRNA-sequencing, we were able to identify circRNAs which were altered by thymoquinone, a natural product with potential anti-cancer activity. Gene enrichment analysis showed that the altered circRNAs were associated with binding of proteins, metal ions, nucleotide, ATP, and RNAs, and correlated with Wnt and Hedgehog signaling pathways (Figure 5), demonstrating the inhibitory effects of thymoquinone on pancreatic cancer. Another group of investigators also utilized circRNA-sequencing to find novel mechanisms of a potential anti-cancer drug nigericin in the treatment of pancreatic cancer [131]. From the profiling data, they found that nigericin could regulate focal adhesion, TNF, MAPK, PI3K-Akt, and HIF-1 signaling pathway, pyrimidine metabolism and purine metabolism through modulation of circRNA-miRNA-mRNA network [131].

### 4.2. Oncogenic circRNAs

#### 4.2.1. ciRS-7

ciRS-7 is an oncogenic circRNA which acts as a sponge of miR-7 [132]. A recent study found that ciRS-7 expression was significantly higher in pancreatic cancer tissues compared to adjacent pancreatic tissues [110]. Moreover, ciRS-7 expression was significantly increased in the area of invasion and metastasis of pancreatic cancer. A mechanistic study showed that knockdown of ciRS-7 inhibited the proliferation and invasion of pancreatic cancer cells with up-regulation of miR-7 through releasing sponge and down-regulation of EGFR and STAT3 pathways by miR-7 [110].

#### 4.2.2. CircEIF6

Oncogenic circRNA circEIF6 (hsa_circ_0060055) was found to be aberrantly up-regulated in pancreatic tumor tissues and cells [111]. Silence of circEIF6 significantly induced apoptosis and inhibited pancreatic cancer cell proliferation, migration, and invasion [111]. A mechanistic study demonstrated that circEIF6 bond to miR-557 led to the up-regulation of SLC7A11 and activation of PI3K/AKT signaling in pancreatic cancer cells [111]. An animal study showed that knockdown of circEIF6 significantly suppressed pancreatic tumor xenograft growth in vivo [111]. These findings suggest that circEIF6 exerts its oncogenic effects through miR-557/SLC7A11/PI3K/AKT signaling transduction.

#### 4.2.3. CircFOXK2

circFOXK2 is an oncogenic circRNA in pancreatic cancer [112]. circFOXK2 was significantly upregulated in pancreatic cancer cells and tissues, leading to increased cell proliferation, migration, and invasion [112]. circFOXK2 acted as a sponge for miR-942, causing the altered expression of downstream target ANK1, GDNF, and PAX6, which could play an oncogenic effect in pancreatic cancer [133]. circFOXK2 also bonded to RNA-binding protein YBX1 and hnRNPK, enhancing the expression of oncogenes NUF2 and PDXK. By interactions with miR-942 and YBX1/hnRNPK, circFOXK2 promoted the progression of pancreatic cancers [112].

#### 4.2.4. circRNA_100782 and circ_001653

A study found that circRNA_100782 was significantly upregulated in pancreatic cancers [117]. Inhibition of circRNA_100782 suppressed cell proliferation and colony formation through downregulation of IL6R and STAT3, which are oncogenes in pancreatic cancer [134]. Luciferase assay showed that miR-124 was a direct target of circRNA_100782. An animal study showed that knockdown of circRNA_100782 inhibited pancreatic cancer xenografts in nude mice [117]. Therefore, it is believed that circRNA_100782 exerts its oncogenic effects through sponging miR-124 to activate IL6/STAT3 signaling [117]. Similarly, circ_001653 was upregulated in pancreatic cancers [135]. Inhibition of hsa_circ_001653 by siRNA suppressed pancreatic cancer cell proliferation and invasion through sponging miR-377, leading to the downregulation of oncogenic HOXC6 expression [135].

#### 4.2.5. hsa_circ_0071036 and hsa_circ_0007534

Recent study showed that hsa_circ_0071036 plays important roles as oncogenic circRNA in tumorigenesis and progression of pancreatic cancers [118]. A mechanistic study showed that hsa_circ_0071036 acted as an efficient sponge for tumor suppressive miR-489 in pancreatic cancer. An in vivo animal study demonstrated that knockdown of hsa_circ_0071036 significantly inhibited pancreatic cancer growth in mice [118]. Similarly, hsa_circ_0007534 was also found to be up-regulated in pancreatic cancer tissues and cells [119]. hsa_circ_0007534 could inhibit pancreatic cancer cell apoptotic death through modulation of Bcl-2/caspase-3 by sponging tumor suppressive miR-625 and miR-892b [119].

#### 4.2.6. circBFAR

circBFAR (hsa_circ_0009065) was found to be upregulated in pancreatic cancer tissues [113]. Moreover, the high expression of circBFAR was correlated with high TNM stage and poor prognosis of patients with pancreatic cancer. Mechanistic study showed that circBFAR promoted expression of MET through sponging tumor suppressive miR-34b-5p, leading to the activation of MET/PI3K/Akt signaling. Importantly, knockdown of circBFAR significantly suppressed pancreatic cancer cell proliferation and motility in vitro and inhibited pancreatic tumor growth and metastasis in mouse model, demonstrating its oncogenic function of circBFAR [113].

#### 4.2.7. circ-ASH2L

circ-ASH2L was highly up-regulated in pancreatic cancer cells and tissues [114]. The high expression of circ-ASHL was positively correlated with lymphatic invasion and TNM stage. A mechanistic study showed that circ-ASH2L induced cell proliferation, tumor invasion, and angiogenesis by sponging miR-34a, which is a tumor suppressive miRNA that suppresses Notch 1. Therefore, by sponging miR-34a, circ-ASH2L enhanced the expression of Notch1, one of the oncogenic signals, leading to tumor progression and poor survival of patients with pancreatic cancer [114].

#### 4.2.8. circRHOT1

circRHOT1 (hsa_circ_0005397) is highly expressed in pancreatic cancer and is mainly located in the cytoplasm of pancreatic cancer cells [115,116]. Down-regulation of circRHOT1 suppressed pancreatic cell proliferation, invasion, and migration. The effects of circRHOT1 could be mediated through sponging miR-26b, miR-125a, miR-330, and miR-382, which could be tumor suppressive miRNAs in pancreatic cancer [116]. A similar study also showed that circRHOT1 served as a sponge and bond to miR-125a-3p, upregulating oncogenic E2F3. Knockdown of circRHOT1 significantly suppressed pancreatic cancer cells through the regulation miR-125a-3p/E2F3 axis [115]. These findings suggest the oncogenic effects of circRHOT1.

### 4.3. Tumor Suppressive circRNAs

#### 4.3.1. circNFIB1

circNFIB1 (hsa_circ_0086375) is a tumor suppressive circRNA in pancreatic cancer [120]. circNFIB1 is downregulated in pancreatic cancer tissues and negatively associated with lymph node metastasis [120]. A study found that knockdown of circNFIB1 enhanced lymph node metastasis of pancreatic cancer both in vitro and in vivo [120]. A mechanistic study showed that circNFIB1 acted as an anti-cancer sponge of oncogenic miR-486-5p. By binding to miR-486-5p, circNFIB1 induced expression of PIK3R1 and, in turn, inhibited the expression of VEGF-C through inactivating PI3K/Akt signaling [120]. An animal study confirmed that circNFIB1 regulated miR-486-5p/PIK3R1/VEGF-C signal axis, leading to the inhibition of lymph node metastasis in pancreatic cancer [120].

#### 4.3.2. hsa_circ_001587

hsa_circ_001587 is another tumor suppressive circRNA in pancreatic cancer [121]. The expression of hsa_circ_001587 was significantly down-regulated in pancreatic cancer tissues and cells [121]. Overexpression of hsa_circRNA_001587 significantly decreased abilities of pancreatic cancer cell proliferation, migration, invasion, angiogenesis and tumorigenesis through inhibition of oncogenic MMP-2, MMP-9, MCM2, and VEGF expression in pancreatic cancer [121]. Mechanistic studies showed that hsa_circRNA_001587 bond to oncogenic miR-223 and, in turn, up-regulated SLC4A4 [121]. Therefore, the tumor suppressive effects of hsa_circ_001587 is mediated through the regulation of miR-223/SLC4A4/MMP signaling pathway.

#### 4.3.3. hsa_circ_0001649

Similarly to other tumor suppressive circRNA, hsa_circ_0001649 was found to be down-regulated in pancreatic cancer tissues and cells [136]. Moreover, the down-regulated hsa_circ_0001649 was associated with the clinical tumor stage of the patients with pancreatic cancer [136]. Study found that overexpression of hsa_circ_0001649 induced apoptotic cell death and inhibited cell proliferation and colony formation in pancreatic cancer cells [136], suggesting the tumor suppressive effects of hsa_circ_0001649 in pancreatic cancer. However, the mechanism underlying hsa_circ_0001649 as a tumor suppressive remains unclear.

## 5. piRNA in Pancreatic Cancer

In recent years, more studies on the roles of piRNAs and PIWI proteins in the development and progression of breast, lung, and gastric cancers have been reported [47,48,50,137,138]. Because piRNAs regulate gene expression and genome stability in stem cells [40,41], any deregulation of piRNAs could cause the development of cancer stem cells and promote cancer progression. A recent study showed that piRNA-651 promoted proliferation and migration of breast cancer cells through epigenetic regulation of PTEN promoter inactivation [48]. It was also found that piRNA-211106 could serve as tumor suppressor to inhibit the progression of lung cancer and enhance the chemotherapy sensitivity of cancer cells [49]. In gastric cancer, piRNA-823 was found to be significantly down-regulated and induction of piRNA-823 inhibited cancer cell growth in vitro and in vivo [139]. These results suggest the importance of piRNAs in cancer. However, the reported research on the roles of piRNA and PIWI proteins in pancreatic cancer is limited.

### 5.1. Genotyping and piRNA Profiling in Pancreatic Cancer

In a recent study on the genetic mechanism of the co-effects of single nucleotide polymorphisms (SNP) and piRNA in cancers, a group of investigators conducted expression quantitative trait locus (eQTL) analysis using data including 10,997 samples across 33 cancer types from The Cancer Genome Atlas (TCGA) [140]. They identified millions of SNP-piRNA pairs in tumor and normal tissues. They also developed a database, piRNA-eQTL, containing eQTL results with differential expression and survival analyses. From the database, it was found that the expression level of piR-317 in pancreatic cancer tissues was lower compared to normal tissue, suggesting its tumor suppressive effect of piR-317 [140]. The expression level of piR-1945036 was significantly up-regulated in pancreatic cancer tissues. The patients with low piR-1945036 had longer survival than the patients with higher piR-1945036 expression [140], suggesting the oncogenic effect of piR-1945036.

### 5.2. Exosome piRNAs in Pancreatic Cancer

Serum exosomes from liquid biopsy are a good source of samples that could be tested for investigation of cell communications. Exosomes in blood circulation contain proteins and RNAs including piRNAs, which could reflect the healthy or pathological conditions of patients [141,142]. In a study on the analysis of exosomes, the exosomes from the serum of healthy people, intraductal papillary mucosal neoplasms, and pancreatic cancer were isolated and total RNAs from exosomes were extracted [141]. The total RNAs were further analyzed by next generation sequencing. Sequencing analysis showed that piRNAs including hsa-piR-52959, hsa-piR-53108, hsa-piR-30690, hsa-piR-54479, and hsa-piR-56621 were up-regulated in patients with pancreatic cancer compared to healthy people [141], suggesting the oncogenic effects of these piRNA. Whereas, several piRNAs such as hsa-piR-54888, hsa-piR-42185, hsa-piR-46410, hsa-piR-58897, and hsa-piR-43043 were down-regulated in patients with pancreatic cancer [141], suggesting that these piRNAs could have tumor suppressive functions. In addition, exosome hsa-piR-001311 and hsa-piR-016658 were found to have abundant level differences between pancreatic cancer and controls [143]. However, the roles of these exosome piRNAs need further investigations.

### 5.3. piR-017061

Next-generation sequencing (NGS) has also been used in the analysis of pancreatic cancer tissues. In a study using NGS, coding RNAs, lncRNAs, and small non-coding RNAs including piRNAs in tissue samples from six pancreatic cancers and five pancreatic controls were analyzed [144]. It was found that piR-017061, a piRNA located within the HBII-296A snoRNA, was significantly downregulated in pancreatic cancer tissues [144]. This result suggests that piR-017061 could be a tumor suppressive piRNA in pancreas. A recent report by another group confirmed the tumor suppressive effects of piR-017061 [44]. They found that the expression of piR-017061 was significantly downregulated in pancreatic cancer tissues and cells. Functional analysis showed that piR-017061 suppressed the growth of pancreatic cancer cell in vitro and in vivo [44]. A mechanistic study identified that EFNA5 mRNA was a target of piR-017061 and that piR-017061 directly bond to EFNA5 mRNA mediated by PIWIL1, leading to the degradation of EFNA5 mRNA. Loss of EFNA5, which had an oncogenic effect, inhibited the development and progression of pancreatic cancer [44]. Therefore, targeting PIWI/piRNA-mediated EFNA5 gene regulation could be a new strategy for the treatment of pancreatic cancer.

## 6. The Role of Non-Coding RNAs in the Diagnosis of Pancreatic Cancer

Several lncRNAs have their tissue-specific and cancer type-specific distribution, suggesting that they can be used as diagnostic biomarkers [145,146,147,148]. Among them, PCA3 (Prostate Cancer Antigen 3) is the most well-recognized lncRNA which can be used for the diagnosis of prostate cancer [148] and is approved by FDA. For diagnosis of pancreatic cancer, no such FDA approved lncRNA-based diagnosis has been reported so far. However, researches on the development of ncRNA testing as diagnosis or supplementary diagnostic tools for pancreatic cancer have received much attention and revealed some novel markers for pancreatic cancer [149,150,151,152].

### 6.1. SNHG15

SNHG15 is an lncRNA which plays critical roles in the development and progression of various cancers through regulation of Akt/mTOR signaling [153,154]. It has been reported as a prognostic marker in colorectal and liver cancers [155,156]. A study was focused on the investigation of SNHG15 as potential diagnostic markers of pancreatic cancer. It was found that the level of SNHG15 was significantly higher in both plasma and cancer tissues from patients with pancreatic cancer [157]. Receiver operator characteristic (ROC) analysis, which is usually used for determining diagnosis ability, showed that plasma SNHG15 could be a biomarker for distinguishing patients with pancreatic cancer from healthy people [157]. Moreover, a high level of SNHG15 was also significantly correlated with lymph node metastasis, higher tumor stage, and shorter overall survival [157], suggesting that SNHG15 is a promising biomarker for the diagnosis and prognosis of pancreatic cancer.

### 6.2. ABHD11-AS1

Circulating lncRNA ABHD11-AS1 has also been identified as a biomarker for the early detection of pancreatic cancer [151]. In a study on lncRNA, 11 pancreatic cancer related lncRNAs were investigated to reveal their roles as diagnostic markers [151]. The levels of these lncRNAs in plasma samples from 114 patients with pancreatic cancer, 97 patients with chronic pancreatitis, and 46 healthy controls were measured and ROC analysis was conducted. It was found that among 11 lncRNAs, ABHD11-AS1 had the best diagnostic performance for the diagnosis of pancreatic cancer [151]. Moreover, ROC analysis suggested that ABHD11-AS1 could be a better biomarker than CEA, CA19-9, and CA125 for early detection of pancreatic cancer. Furthermore, it was found that the combination of ABHD11-AS1 and CA19-9 was more efficient for the diagnosis of pancreatic cancer [151], suggesting the importance of the new diagnostic marker ABHD11-AS1.

### 6.3. HULC

HULC is a lncRNA that promotes cancer cell EMT, invasion, and metastasis [158,159]. The roles of HULC as a biomarker in the diagnosis of pancreatic cancer have been investigated. The study found that extracellular vesicle encapsulated HULC in blood could be a potential biomarker for the diagnosis of pancreatic cancer [150]. HULC level was significantly higher in the patients with pancreatic cancer compared to healthy individuals [150]. Further analysis showed that HULC in blood extracellular vesicle had good predictive performance for diagnosis of pancreatic cancer. In addition, another separate study using blood samples from 60 patients with pancreatic cancer and 60 patients with benign pancreatic diseases showed that high expression of HULC in serum could be used to distinguish patients with pancreatic cancer from patients with benign pancreatic diseases [149]. Interestingly, HULC could serve as a serum biomarker for the diagnosis and prognosis of gastric cancer [160]. These findings suggest that lncRNA HULC could be a novel biomarker for the diagnosis and prognosis of pancreatic and gastric cancers.

### 6.4. UFC1

The lncRNA UFC1 has been known as an oncogenic lncRNA regulating EZH2 signaling in cancer [161]. The role of serum UFC1 in the diagnosis of pancreatic cancer was investigated. Compared to healthy subjects, the level of serum UFC1 in pancreatic cancer patients was significantly higher [162]. ROC analysis showed that serum UFC1 levels could effectively distinguish patients with pancreatic cancer from healthy subjects. Moreover, the serum UFC1 level was correlated with lymph node metastasis, distant metastasis, and clinical stage [162]. The patients with high level of UFC1 had shorter progression-free and overall survival than those with a low level of UFC1 [162]. These results clearly suggest that serum lncRNA UFC1 could be a novel biomarker for the diagnosis and prognosis of pancreatic cancer.

### 6.5. circ_001569

circ_001569 is an oncogenic circRNA regulating PI3K/Akt and Wnt signaling [163]. High expression of circ_001569 have been found in colorectal and liver cancers [164,165,166]. To find the diagnostic and prognostic values of circ_001569 in pancreatic cancer, the expression levels of circ_001569 in plasma samples from 71 patients with pancreatic cancer and 71 healthy subjects were measured [167]. It was found that the level of plasma circ_001569 was significantly increased in patients with pancreatic cancer [167]. ROC analysis showed that plasma circ_001569 could efficiently distinguish patients with pancreatic cancer from healthy subjects. In addition, the high level of circ_001569 was positively associated with lymph node metastasis, tumor stage, and invasion [167]. The pancreatic cancer patients with a high level of circ_001569 had a poor prognosis. These observations suggest that circ_001569 in plasma could be a promising biomarker for the diagnosis and prognosis of pancreatic cancers.

### 6.6. circ-LDLRAD3

circ-LDLRAD3 is another circRNA which could be used for the diagnosis of pancreatic cancer. In a study on circ-LDLRAD3, the expression levels of circ-LDLRAD3 in pancreatic cancer and adjacent non-tumorous tissues, plasma samples from patients with pancreatic cancer and plasma samples from healthy subjects were measured [168]. It was found that the level of circ-LDLRAD3 was significantly increased in pancreatic cancer tissues and plasma compared to normal tissues or plasma [168]. ROC analysis showed that circ-LDLRAD3 alone could efficiently diagnose pancreatic cancer [168]. A combination of circ-LDLRAD3 with CA19-9 had a much higher diagnostic sensitivity and specificity for pancreatic cancer. In addition, the high level of circ-LDLRAD3 was also significantly correlated with invasion and metastasis pancreatic cancer [168]. These results demonstrate that the level of circ-LDLRAD3 could be used as diagnostic and prognostic markers for pancreatic cancer.

## 7. The Role of Non-Coding RNAs in the Therapy of Pancreatic Cancer

In the development of cancer therapy targeting ncRNAs, strategies for the delivery of anti-ncRNA molecules include nanoparticle-packed siRNA delivery, LNA GapmeRs delivery and exosome-based delivery [169,170,171]. Nanoparticle-packed siRNA has been utilized to inhibit lncRNAs in clinical trial [169]. LNA GapmeRs delivery system has been successfully used to inhibit lncRNA MALAT1 [170]. Studies have also confirmed that exosome can deliver exogenous RNAs including siRNAs and other ncRNAs [171,172,173,174]. Therefore, exosome-based delivery of siRNAs and other ncRNAs could be another novel strategy for the treatment of cancers.

ncRNAs also play important roles in drug resistance and the prognosis of pancreatic cancer [175,176,177,178]. In the treatment of pancreatic cancer, ncRNAs could influence the efficacy of cancer therapeutics including chemotherapy, radiotherapy, immunotherapy, and other targeted therapy by regulation of cancer cell sensitivity to therapies. In addition, some ncRNAs could be used for predicting the prognosis of pancreatic cancer patients after treatment.

### 7.1. ncRNAs Regulated Drug Sensitivity in Pancreatic Cancer

Gemcitabine resistance is a major chemoresistance in the treatment of pancreatic cancer. A study was conducted to find the difference of lncRNA profiles between gemcitabine sensitive and resistant pancreatic cancer cells using genomic analysis [178]. By comparing lncRNA profiles between gemcitabine sensitive and resistant cells, it was found that 4983 of 13,310 detected lncRNAs demonstrated more than 2-fold changes, suggesting that lncRNA regulation is significantly involved in drug resistance in pancreatic cancer [178]. PVT1 is a lncRNA and significantly overexpressed in pancreatic cancer [79]. PVT1 has been identified as a regulator of gemcitabine sensitivity. Study showed that functional inactivation of PVT1 resulted in enhanced gemcitabine sensitivity whereas overexpression of PVT1 enhanced resistance to gemcitabine in human pancreatic cancer [79]. Another lncRNA HOTTIP is also significantly upregulated in human pancreatic cancer [83]. High expression of HOTTIP in pancreatic cancer induced gemcitabine resistance while inhibition of HOTTIP potentiated anticancer activity of gemcitabine in vitro and in vivo [83]. In addition, linc-ROR is also a lncRNA which is up-regulated in pancreatic cancer [179]. Linc-ROR was also involved in the regulation of gemcitabine sensitivity in pancreatic cancer [180]. Down-regulation of linc-ROR significantly sensitized pancreatic cancer cells to gemcitabine whereas overexpression of linc-ROR significantly reduced the sensitivity of pancreatic cancer cells to gemcitabine [180]. lncRNA MALAT-1 is also associated with chemoresistance of pancreatic cancer [60]. A study showed that an elevated level of MALAT-1 decreased sensitivity to gemcitabine in pancreatic cancer cells [60], suggesting that down-regulation of MALAT-1 could increase the sensitivity of pancreatic cancer cells to gemcitabine.

In addition to lncRNAs, circRNAs also regulate the sensitivity of pancreatic cancer to gemcitabine [176,181]. A study showed that silencing two circRNAs (chr14:101402109-101464448+ and chr4:52729603-52780244+) in gemcitabine resistant pancreatic cancer cells restored sensitivity to gemcitabine, suggesting the role of these circRNAs in the regulation of gemcitabine resistance [176]. By profiling circRNAs in gemcitabine resistant and sensitive pancreatic cancer cells, it was found that circ_101672, circ_004077, and circ_003251 were the top three upregulated circRNAs while circ_101543, circ_102747, and circ_000926 were the top three down-regulated circRNAs in gemcitabine resistant pancreatic cancers [182], suggesting that regulation of these circRNAs could restore the sensitivity of pancreatic cancer cells to gemcitabine. CircHIPK3 is another circRNA up-regulated in gemcitabine resistant pancreatic cancer cells and tumor tissues [181]. Knockdown of circHIPK3 in PANC-1-GEM and SW-1990-GEM gemcitabine resistant cells could significantly inhibit cell proliferation, invasion, migration, and sensitize pancreatic cancer cells to gemcitabine [181].

Since ncRNAs play important roles in the chemoresistance of pancreatic cancer, therapies targeting ncRNAs could reduce chemoresistance in pancreatic cancer. Therefore, therapeutic strategy targeting ncRNAs could be designed as to block chemoresistance related ncRNAs by siRNAs. Because the short nucleotides such as siRNAs are easy to be degraded in vivo, novel efficient delivery systems are needed for successful delivery of short nucleotides into target cells [183]. Recently, nanoparticle delivery systems have been used for delivery of ncRNAs to pancreatic cancer cells and tumor microenvironments for the treatment of pancreatic cancer [184,185].

### 7.2. ncRNAs for the Prognosis of Pancreatic Cancer

Recent studies have demonstrated that certain lncRNAs could be used for the prognosis of pancreatic cancer [53,186,187]. A study on 80 patients with pancreatic cancer showed that high expression of lncRNA AFAP1-AS1 and UCA1, and low expression of ENSG00000218510 could predict poor overall survival and tumor progression in pancreatic cancer [186]. Similar results from another group also demonstrated that AFAP1-AS1 overexpression was associated with lymph node metastasis, perineural invasion, and poor survival [187]. The study also confirmed that knockdown of AFAP1-AS1 led to the inhibition of pancreatic cancer cell proliferation, migration, and invasion, demonstrating the value of AFAP1-AS1 as a prognosis marker [187]. HOTAIR is another lncRNA which is highly expressed in pancreatic cancer [55]. HOTAIR could be another prognostic markers for pancreatic cancer. A study showed that patients with low HOTAIR expression had significantly increased overall survival compared to patients with high HOTAIR expression [53]. As mention early, MALAT1 is another oncogenic lncRNA highly expressed in pancreatic cancer [63]. Clinical studies demonstrated that MALAT1 was correlated with clinical progression and unfavorable prognosis in pancreatic cancer and that patients with higher expression of MALAT1 had a poorer disease free survival compared to patients with low expression of MALAT1 [63,100]. In a study on lncRNA profiling analysis with clinical data, it was found that the expression level of lncRNA BC008363 was significantly lower in pancreatic cancer tissues compared to normal pancreatic tissues [175]. More importantly, patients with high expression level of BC008363 had significantly better survival than those with low expression level of BC00836 [175], suggesting its value as a prognostic marker. In addition, oncogenic lncRNA PVT1 was found to be an independent prognostic factor for poor overall survival in patients with pancreatic cancer [73]. Pancreatic cancer patients with high expression of PVT1 had shorter overall survival times compared to those with low expression of PVT1 [73].

In addition to lncRNAs, circRNAs could also function as prognostic markers. circ-ADAM9 is an oncogenic circRNA [188]. In a study on 58 patients with pancreatic cancer, high expression of circ-ADAM9 was found in pancreatic cancer tissues with advanced clinical stage and lymph node metastasis [188]. More importantly, it was found that pancreatic cancer patients with high expression of circ-ADAM9 had poorer survival rate than those with low expression of circ-ADAM9 [188]. Another circRNA circ-LDLRAD3 was shown to be overexpressed in pancreatic cancer tissues and cells [168,189]. Further analysis showed that high expression of circ-LDLRAD3 was a marker indicating a poor prognosis in patients with pancreatic cancer [189]. CircRNA circ_0030235 was also significantly increased in pancreatic cancer tissues and cells compared to normal pancreatic tissues and cells [177]. Clinical study found that overexpression of circ_0030235 in tumor samples was associated with higher tumor stage and lymph node invasion [177]. Kaplan-Meier analysis showed that high expression of circ_0030235 significantly predicted poor prognosis in pancreatic cancer [177]. circRNA hsa_circ_0001649 could also serve as an independent prognostic biomarker of pancreatic cancer [136]. Fisher’s exact test found that patients with low expression of hsa_circ_0001649 had an advanced tumor stage and lower differentiation grade [136]. Kaplan-Meier analysis showed that the patients with high expression of hsa_circ_0001649 had better 5-year overall survival [136], suggesting the prognostic value of hsa_circ_0001649. In addition, testing circRNA in plasma samples to predict the prognosis of patients with pancreatic cancer is a more convenient way in clinic. Studies found that high level of circ-IARS or circ-PDE8A in plasma exosomes secreted by pancreatic cancer indicated a poor overall survival of patients with pancreatic cancer [190,191]. These findings on ncRNAs could help to design targeted therapeutic strategy more optimally to better treat pancreatic cancers.

## 8. Conclusions

Pancreatic cancer is the seventh leading cause of cancer related deaths worldwide. In the United States [192], pancreatic cancer remains the fourth leading cause of cancer related death with an estimated 60,430 new cases and 48,220 deaths in 2021 in the United States [193]. It is expected that pancreatic cancer will become the second leading cause of cancer-related death by 2030 [194]. Both incidence and mortality rates for pancreatic cancer have increased since 2000 in US [193]. There are no early screening biomarkers for pancreatic cancer. Currently, CA19-9 is a biomarker for the diagnosis and prognosis of pancreatic cancer. However, CA19-9 has limitation in the early diagnosis of this disease because of its false positivity and nonspecific characteristics [195]. Therefore, there is an urgent need to find novel and effective biomarkers for the diagnosis of pancreatic cancer. Moreover, so far there are no specific or effective drugs to overcome chemoresistance for the treatment of pancreatic cancer. Thus, finding novel therapeutic and prognostic biomarkers is critically important for the improvement of cancer therapy for pancreatic cancer.

The recent studies described above indicate that ncRNAs including lncRNAs, circRNAs, and piRNAs play critical roles in the regulation of the development and progression of pancreatic cancer. Therefore, these ncRNAs could be novel biomarkers for the diagnosis and treatment of pancreatic cancer. For the diagnosis of pancreatic cancer, ncRNAs in extracellular vesicles such as SNHG15, ABHD11-AS1, HULC, UFC1, circ_001569, and circ-LDLRAD3 described above could serve as diagnostic biomarkers combined with CA19-9 in liquid biopsy for better diagnosis of pancreatic cancer. This non-invasive biopsy is more convenient in clinic. For the treatment of pancreatic cancer, currently available studies have shown that several ncRNAs have effects on the regulation of drug sensitivity in pancreatic cancer. These ncRNAs include PVT1, HOTTIP, linc-ROR, MALAT-1, circHIPK3, circ_101672, circ_004077, circ_003251, circ_101543, circ_102747, and circ_000926. Therefore, regulations of these ncRNAs could increase pancreatic cancer cell sensitivity to conventional therapies to achieve better treatment outcome. In addition, several ncRNAs such as AFAP1-AS1, UCA1, HOTAIR, PVT1, MALAT-1, circ-ADAM9, BC008363, circ-LDLRAD3, circ-IARS, circ-PDE8A, circ_0030235, and circ_0001649 have been associated with prognosis of pancreatic cancer. Detection of these ncRNAs could predict the treatment outcome in pancreatic cancer. Targeting ncRNAs by siRNA is main strategy for targeted therapy [172,173]. However, easy degradation and difficult delivery of siRNA are challenges for ncRNA targeted therapy. Nevertheless, using nanoparticle-packed siRNA, LNA GapmeRs, and exosome-based delivery to protect siRNA and target oncogenic ncRNAs is on its way and will be tested in pre-clinical and clinical studies. It is important to note that more molecular studies and in vivo pre-clinical studies are needed before these strategies targeting ncRNAs could be used in clinical practice.

## Figures and Tables

**Table 1:** Major lncRNAs in pancreatic cancer.

lncRNA	Role	Functions	Related Signaling	Ref.
HOTAIR	Oncogenic. Up-regulated in cancer.	Promote proliferation and drug resistance.	EZH2, miR-34a.	[52,53,54,55,56,57]
MALAT-1	Oncogenic. Up-regulated in cancer.	Promote cell growth, migration, invasion and metastasis.	Sox-2, EZH2, miR-216a, miR-217, miR-200c, Hippo-YAP.	[58,59,60,61,62,63,64,65,66]
H19	Oncogenic. Up-regulated in cancer.	Promote cell proliferation, tumor growth and metastasis.	let-7, HMGA2, E2F, miR-675, miR194.	[67,68,69,70,71,72]
PVT1	Oncogenic. Up-regulated in cancer.	Promote cell proliferation, migration, drug resistance.	p21, miR-448, miR-20a-5p, ULK1, miR519, HIF-1, YKT6, RAB7, VAMP3.	[73,74,75,76,77,78,79,80,81]
HOTTIP	Oncogenic. Up-regulated in cancer.	Promote cell growth, invasiveness and drug resistance. Modulate stem cells.	HOXA9, miR-137, HOXA13.	[82,83,84,85]
GAS5	Tumor suppressive. Down-regulated in cancer.	Reverse EMT, inhibit metastasis and increase drug sensitivity.	CDK6, miR-221/SOCS3, miR-32, miR-181c.	[86,87,88,89]
MEG	Tumor suppressive. Down-regulated in cancer.	Inhibit cell proliferation, migration and invasion.	c-Met, PI3K/AKT.	[90,91,92,93,94]

**Table 2:** Major circRNAs in pancreatic cancer.

circRNA	Role in Pancreatic Cancer	Functions	Related miRNA and Signaling	Ref.
ciRS-7	Oncogenic. Up-regulated in cancer.	Promote cell proliferation and invasion.	Sponge miR-7. Regulate EGFR and STAT3.	[110]
circEIF6	Oncogenic. Up-regulated in cancer.	Promote cell proliferation and inhibit apoptosis.	Sponge miR-557. Regulate SLC7A11 and PI3K/AKT.	[111]
circFOXK2	Oncogenic. Up-regulated in cancer.	Promote cell proliferation, migration and invasion.	Sponge miR-942. Regulate ANK1, GDNF, PAX6, NUF2 and PDXK	[112]
circBFAR	Oncogenic. Up-regulated in cancer.	Promote cell proliferation and motility.	Sponge miR-34b-5p. Regulate MET/PI3K/Akt.	[113]
circ-ASH2L	Oncogenic. Up-regulated in cancer.	Induce cell proliferation, tumor invasion and angiogenesis.	Sponge miR-34a. Regulate Notch 1.	[114]
circRHOT1	Oncogenic. Up-regulated in cancer.	Promote cell proliferation, migration and invasion.	Sponge miR-125a, miR-330, miR-26b and miR-382. Regulate E2F3.	[115,116]
circRNA_100782	Oncogenic. Up-regulated in cancer.	Promote cell proliferation and tumor growth.	Sponge miR-124. Regulate IL6 and STAT3.	[117]
hsa_circ_0071036	Oncogenic. Up-regulated in cancer.	Promote cell proliferation, invasion and tumor growth.	Sponge miR-489.	[118]
hsa_circ_0007534	Oncogenic. Up-regulated in cancer.	Inhibit apoptosis.	Sponge miR-625 and miR-892b. Regulate Bcl-2 and caspase-3.	[119]
circNFIB1	Tumor suppressor. Down-regulated in cancer.	Inhibit lymph node metastasis.	Sponge miR-486-5p. Regulate PIK3R1 and VEGF-C.	[120]
hsa_circ_001587	Tumor suppressor. Down-regulated in cancer.	Inhibit cell proliferation, migration, invasion and angiogenesis.	Sponge miR-223. Regulate SLC4A4, MMP-2, MMP-9, MCM2 and VEGF.	[121]
